# Non-thermal plasma with 2-deoxy-D-glucose synergistically induces cell death by targeting glycolysis in blood cancer cells

**DOI:** 10.1038/srep08726

**Published:** 2015-03-04

**Authors:** Neha Kaushik, Su Jae Lee, Tae Gyu Choi, Ku Youn Baik, Han Sup Uhm, Chung Hyeok Kim, Nagendra Kumar Kaushik, Eun Ha Choi

**Affiliations:** 1 Plasma Bioscience Research Center, Kwangwoon University, Seoul 139-701, Korea; 2 Laboratory of Molecular Biochemistry, Department of Life Science, Hanyang University, Seoul 133-791, Korea; 3 School of medicine, Department of Biochemistry and Molecular Biology, Kyunghee University, Seoul 130-701, Korea; 4 Institute of Information Technology, Kwangwoon University, Seoul 139-701, Korea

## Abstract

In this study, we show the selective and efficient anti-cancer effects of plasma (at a low dose) when cell metabolic modifiers are also included. 2-deoxy-D-glucose (2-DG), a glycolytic inhibitor, was used with effective doses of non-thermal plasma, synergistically attenuating cell metabolic viability and inducing caspase-dependent and independent cell death. The combination treatment decreased the intracellular ATP and lactate production in various types of blood cancer cells *in vitro*. Taken together, our findings suggest that 2-DG enhances the efficacy and selectivity of plasma and induces the synergistic inhibition of cancer cell growth by targeting glycolysis and apoptosis. Specifically, this treatment strategy demonstrated an enhanced growth inhibitory effect of plasma in the presence of a metabolic modifier that was selective against cancer cells, not non-malignant cells. This is the first study to report the advantage of combining plasma with 2-DG to eradicate blood cancer cells. Finally, we conclude that 2-DG with non-thermal plasma may be used as a combination treatment against blood cancer cells.

Selectivity, or the preferential, efficient killing of cancer cells without adverse toxicity to normal cells, is one of the most important considerations in cancer treatment. Non-malignant and cancer cells vary distinctly in their energy metabolism. Increased aerobic glycolysis in tumor cells were first identified by Otto Warburg^1^. Based on his experimental data, he revealed that cancer cells (transformed cells) demonstrate a significantly higher glycolysis rate than healthy cells, even in the presence of adequate oxygen, and considered this event to be the most fundamental metabolic alteration in malignant transformation, or ‘the origin of cancer cells’^2^. Cancer cells grow quickly and require energy production that favors this rapid growth. In cancer cells, this glycolysis pathway is not as efficient as oxidative phosphorylation (OXPHOS) at producing energy^3^,^4^. Therefore, to meet their high-energy demands, tumor cells noticeably increase the rate of glycolysis^5^,^6^. Glucose is obtained by increasing the expression of glucose transporters, GLUTs, at the cell membrane^7^,^8^. The dependency of cancer cells on glycolysis for energy production is currently considered as novel pathway for targeting in cancer treatment^9^,^10^. 2-deoxy-D-glucose (2-DG) is a well-characterized metabolic modifier that inhibits the tumor glycolysis pathway. It causes cytotoxicity in tumor cells by disrupting thiol metabolism. 2-DG is glucose analog^11^ in which the 2-hydroxyl group is replaced with hydrogen. Briefly, 2-DG forms 2-deoxy-glucose 6-phosphate through hexokinase phosphorylation. This substrate cannot be used in glycolysis, is eventually trapped in the cytoplasm and becomes an inhibitor of the glycolysis pathway. Subsequently, the cellular energy ATP levels are reduced, resulting in a weak cancer cell. Several findings from previous reports suggest that 2-DG mimics the effects of glucose deprivation and can be successfully used alone or in combination with other drugs in cancer treatments^12^,^13^. Because 2-DG primarily accumulates in cancer cells and partially inhibits the highly utilized glycolysis in these cells, the administration of 2-DG is a safe and effective way of slowing cancer growth^7^.

The application of plasma medicine technology has been actively explored over the last several years. Recently, non-thermal plasmas have demonstrated potential as a safe anticancer therapeutic approach that can kill various types of cancer targets, such as leukemia^14^, lung^15^, glioma^16^,^17^, and melanoma^18^,^19^ cancers. In particular, the accumulating evidence indicates that reactive oxygen species (ROS) play a major role in plasma-induced apoptosis *in vitro*^20^,^21^,^22^,^23^,^24^,^16^. Higher ROS levels induce oxidative stress and directly attack DNA, protein, and other cellular components, finally contributing to apoptosis induction^25^. However, targeting metabolism with non-thermal plasma remains a relatively unexplored area of research. Our earlier study showed that a non-thermal plasma jet could result in a ROS-dependent cell death in leukemia^26^. In an attempt to further determine whether plasma can be more selective and efficient for killing blood cancer cells, we combined 2-DG with a plasma treatment, which contributed to plasma-induced apoptosis, in the present study. We propose that cancer cells were more sensitive than their normal counterparts when treated with a 2-DG and plasma combination treatment. The findings suggest that the inhibition of glycolysis may be a potentially effective strategy for targeting blood cancer cells *in vitro*.

## Results

### Non-thermal plasma jet treatment and the proposed experimental plan

**Figure 1:**
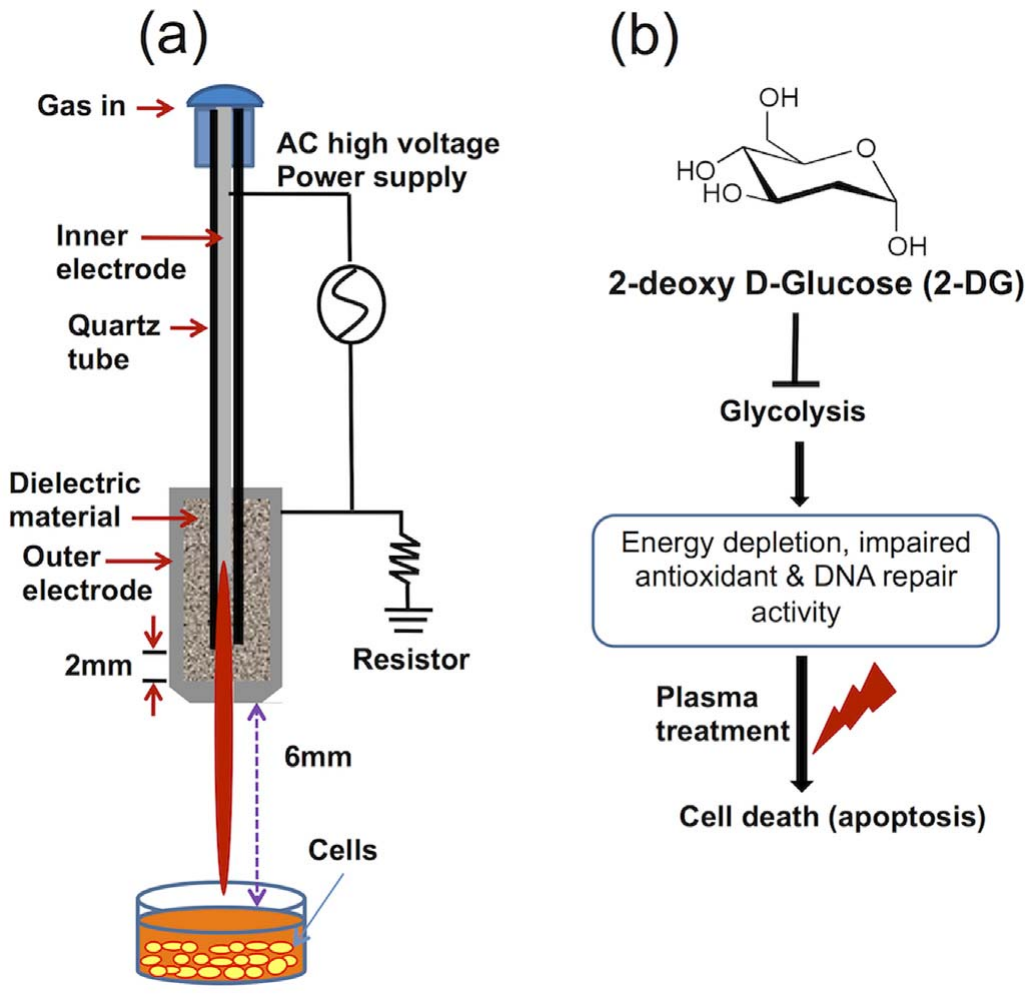
Non-thermal plasma jet and proposed experimental plan (a) Schematic representation of the non-thermal plasma jet system (b) the proposed experimental plan to treat the blood cancer cells using 2-deoxy-D-glucose (2-DG) and plasma.

Figure 1a shows a schematic configuration of the soft air jet plasma system at atmospheric pressure, which primarily consists of a high-voltage power supply, electrodes, and dielectrics. The porous stone has a porosity of 30% and a pore diameter of 150 μm; it acts, as a dielectric vehicle between stainless steel electrodes to produce micro-discharges and reduce the gas temperature. The output voltage (2 kV) and current (13 mA) waveforms have a profile with an average power of 2.6 W (supporting information, Figure S1). The air gas flow rate remained constant at approximately 1 Litre/minute (l/min), and the system was operated in ambient environment with across a 1 mm gas hole throughout the experiment. Optical emission spectroscopic (OES) measurements were obtained using a charge-coupled device spectrometer (HR4000, Ocean Optics, Dunedin, FL, USA) (supporting information, Figure S2). OES was carried out to evidence the spectral line of excited state species in plasma. Fig. S2 is the typical emission spectra (275–950 nm) of the plasma emitted by the air jet plasma. Large number of excited species such as OH**·**, N_2_^+^, and O were observed in OES. These excited species could interact as soon as being emitted, can also form NO and other active species^22^. It is also mentioned that species at the wavelengths of 316, 337, 358 nm could be defined as N_2_^3^Π or NO β ^2^Π (denoted as N_2_/NO), because both of the two species have possible optical emission at these wavelengths^23^. Earlier reports also suggested that plasma stimulated reactive nitrogen species (RNS) play a role in cancer treatments^22^,^23^,^24^ and increase in the intracellular reactive nitrogen species generated or stimulated by plasma also leads to the increase of the intracellular ROS concentration^21^. The gas temperature of the plasma device was around 450 K from N_2_ rotational spectra 340–360 nm of air (supporting information, Figure S3). We also outlined our proposed experimental schematic plan in Figure 1b.

### 2-DG and plasma treatment synergistically suppress the growth of cancer cells

**Figure 2:**
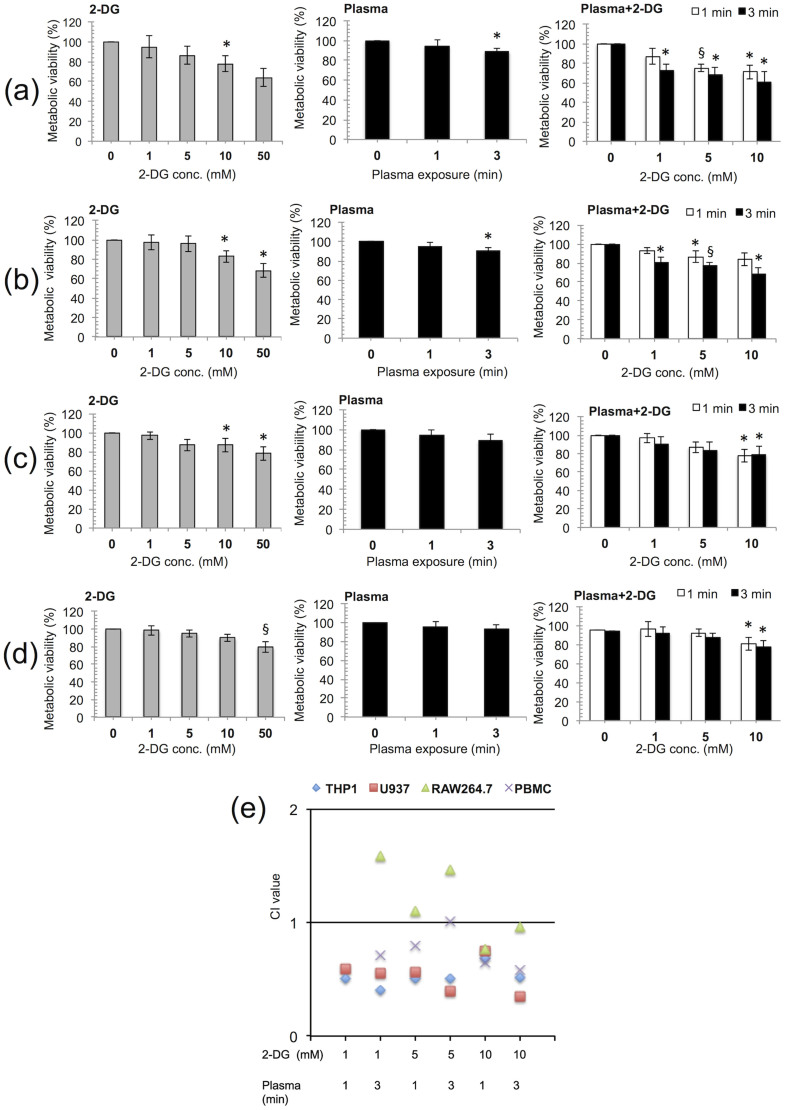
Plasma in combination with 2-deoxy-D-glucose (2-DG) inhibit the growth of blood cancer cells. 2-DG was added 4 hours (h) before plasma treatment and the medium was changed during the experiment. We measured the metabolic viability of (a) THP-1 (human leukemic) cells, (b) U937 (human monocyte lymphoma) cells, (c) RAW264.7 (mouse leukemic) cells and (d) PBMCs (normal blood mononuclear cells) by 2-DG alone, plasma alone and 2-DG + plasma respectively, after 24 h incubation. (e) The combination index (CI) value of 2-DG, plasma and combined treatments in THP-1, U937, RAW264.7 and PBMCs cells were calculated using the Chou-Talalay method. The results were calculated as the percentage of viable cells and presented as the mean ± SD (n = 3). Student's *t*-test was performed, and the significance is indicated as * *p* < 0.05, § *p* < 0.01, and # *p* < 0.001.

Plasma has been shown to cause programmed cell death (apoptosis) in many blood cancer cell lines^27^,^28^. To determine whether plasma can more selectively and efficiently induce apoptosis at a low dose when used in combination with 2-DG (a glycolysis inhibitor)^29^, two human and one-mouse leukemic cancer cell lines THP-1, U937, and RAW264.7, were treated with plasma in the presence of various concentrations of 2-DG. Representative cell metabolic viabilities from all of these cell lines are shown in Figure 2. The inhibitory effect of 2-DG alone was not significant (*p* > 0.05) at the 1 and 5 mM doses, but the 10 and 50 mM 2-DG doses significantly (*p* < 0.05) decreased the cell viability in all of the blood cancer cells tested (Figure 2, supporting information, Figure S4 and S5). As shown in Figure 2a and 2b, the viability of the THP-1 and U937 cells was significantly (*p* < 0.05) reduced by the 2-DG and plasma combination (1 mM 2-DG and 3 min plasma) treatment. A combination treatment (1 mM 2-DG and 3 min plasma) resulted in approximately 19%–27% inhibition of cell growth in THP-1 and U937, which was significant (*p* < 0.05). At higher doses (10 mM 2-DG, 3 min plasma), 32%–49% growth inhibition was observed in both types of cells at all incubation times (Figure 2a and 2b, supporting information, Figure S6 and S7). However, the RAW264.7 cells were found to be the least sensitive to the combination treatments at all doses compared with the THP-1 and U937 cells (Figure 2c, supporting information, Figure S6 and S7). In the case of normal mononuclear cells (PBMCs), no significant (*p* > 0.056) inhibitory effect was observed following combination treatments up to 5 mM 2-DG and 3 min plasma (Figure 2d, supporting information, Figure S7). Among all the blood cells tested, the THP-1 and U937 cells were the most sensitive to the growth-inhibitory effects of the combination treatment (Figure 2a and 2b, supporting information, Figure S6). The cell viability experiments results indicate that the 2-DG and plasma combination treatment inhibits human blood cancer cell growth, which may be due to apoptotic cell death. To further study the synergistic effect of plasma and 2-DG, the entire range of fraction-affected values was calculated as previously described by Chou and Talalay^30^,^31^. Figure 2e and supporting information, Table S1 quantitatively describes the synergistic effect of 2-DG and plasma. The combination index is lower than 1, suggesting that there is synergism with all the 2-DG and plasma combination treatments in THP-1 and U937 cells (CI < 0.77).

### 2-DG and plasma induces cancer cell metabolic alterations

**Figure 3:**
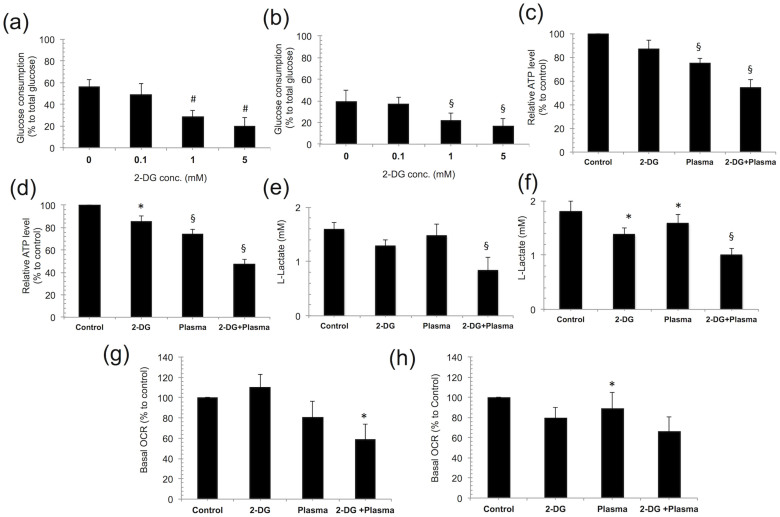
2-deoxy-D-glucose (2-DG) and plasma induces cancer cell metabolic alterations. (a, b) Glucose consumption was examined at various concentrations of 2-DG (0.1, 1, and 5 mM) in THP-1 and U937 cells cultured in RPMI-1640 medium, respectively. (c, d) Cellular ATP levels were measured in THP-1 and U937 cells following a 2-DG (1 mM) and plasma (3 min) combination treatment, respectively. (e, f) Lactate production was monitored following a combination treatment in THP-1 and U937 cells cultured in RPMI-1640 medium, respectively. The oxygen consumption rate (OCR) in THP-1 and U937 cells following a 24 h combination treatment were determined using a seahorse XF24 analyzer. (g, h) The basal level of the OCR following treatment represents the oxidative phosphorylation (OXPHOS) activity in the THP-1 and U937 cancer cells respectively. Error bars designate the ± SD (n = 3). Student's *t*-test was performed, and the significance is indicated as * *p* < 0.05, § *p* < 0.01, and # *p* < 0.001.

To investigate whether 2-DG and plasma regulate the mitochondrial metabolic behavior in cancer cells, we first examined glucose consumption and intracellular ATP and lactate production in blood cancer cells following a combination treatment. Glucose consumption significantly (*p* < 0.01) decreased in THP-1, U937 (Figure 3a and 3b) and RAW264.7 cells (supporting information, Figure S8a) after the 1 and 5 mM 2-DG treatments. Note that this effect was highly significant (*p* < 0.001) in THP-1 cells. However, glucose consumption in the PBMCs was less affected up to the 5 mM 2-DG treatment (supporting information, Figure S8b). We also observed that intracellular ATP and lactic acid production were significantly decreased at 24 hour (h) after combination treatment in all the blood cancer cell lines. We found that the ATP level was significantly affected after the 2-DG and plasma treatments alone but the combined treatment (1 mM 2-DG and 3 min plasma) caused a drastic reduction in ATP by 24 h, 45% (*p* = 0.007) and 52% (*p* = 0.001 highly significant), in the THP-1 and U937 blood cancer cell lines, respectively (Figure 3c and 3d). However, in the RAW264.7 cells, the decrease in the ATP level was the least significant (*p* = 0.045) compared with the untreated control (supporting information, Figure S8c). Normal PBMCs were also less affected with regard to the intracellular ATP decrease, which was not significant (*p* = 0.09) (supporting information, Figure S8d). A similar profile for lactic acid production was also observed in THP-1 and U937 blood cancer cell lines. We found that lactate production was significantly decreased in the THP-1 (*p* = 0.007) and U937 (*p* = 0.002) cells (Figure 3e and 3f) by the combination treatment and that the change in lactate production was less severe in the RAW264.7 cells (supporting information, Figure S8e) than in the control. Nevertheless, lactate production was least affected by the combination treatment in the PBMCs (supporting information, Figure S8f). These findings indicate that the combination treatment plays different roles in regulating mitochondrial metabolism in the different types of blood cells. To support this proposal, we measured the oxygen consumption rate (OCR), an indicator of OXPHOS, in the THP-1 and U937 cells (Figure 3g and 3h). Both cell types showed a markedly significant decrease (*p* < 0.05) in the basal OCR after a 24 h combination treatment compared with the untreated control, and not surprisingly, we also observed a significant decrease (*p* < 0.05) in the ECAR (extracellular acidic ratio; an indicator of glycolysis flux) following a combination treatment in both cell types (supporting information, Figure S9). Together, these results demonstrate that the plasma and 2-DG combination treatment alters mitochondrial energy metabolism by controlling glucose metabolism from glycolysis to OXPHOS in glucose-addicted cancer cells.

### Cancer cells undergo ROS-mediated caspase-dependent apoptosis exclusively through the PARP cleavage induction

**Figure 4:**
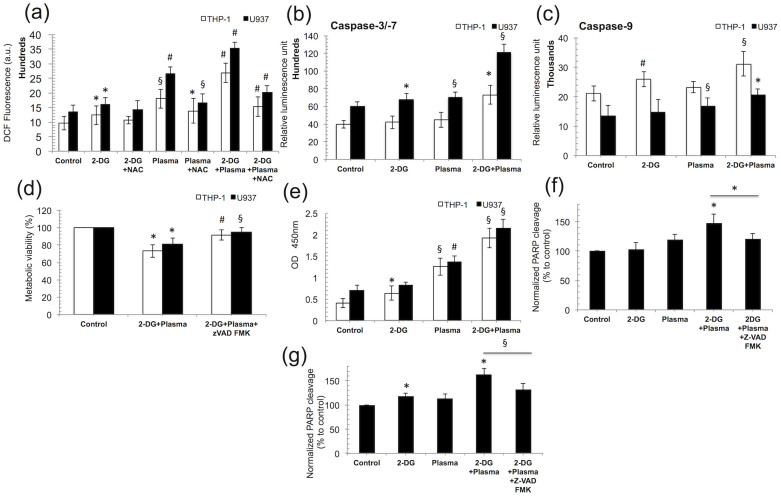
Cancer cells undergo ROS-mediated caspase-dependent apoptosis entirely following PARP cleavage induction by the combination treatment (1 mM 2-DG and 3 min plasma). (a) The intracellular ROS level was measured in THP-1 and U937 cells with N-acetyl L-cysteine (NAC), ROS inhibitor using 10 μM 2′,-7-dichlorodihydrofluorescein diacetate (H_2_DCFDA; Molecular Probes) for 30 min and the DCF fluorescence was quantified at 485/540 nm using a plate reader. (b) The caspase −3/−7 and (c) caspase-9 activities were measured in THP-1 and U937 cells and the luminescence was measured using a plate reader. (d) The metabolic viability of THP-1 and U937 cells was measured using the MTS assay in presence or absence of the pan-caspase inhibitor Z-VAD FMK (100 μM). (e) Quantification of 8-hydroxy-2′ -deoxyguanosine (8-OHdG), a DNA damage marker, in THP-1 and U937 cells exposed to combination treatment. (f, g) The relative percentage level of PARP-1 cleavage in THP-1 and U-937 cells was determined using an ELISA-based method, respectively. The cells were pre-incubated with the inhibitor Z-VAD FMK (100 μM) for 2 h before the combination treatment (1 mM 2-DG and 3 min plasma). Error bars designate the ± SD (n = 3). Student's *t*-test was performed, and the significance is indicated as * *p* < 0.05, § *p* < 0.01, and # *p* < 0.001.

Plasma has been shown to induce oxidative stress, i.e., generate ROS in various cancer cells to activate apoptosis signaling cascades^32^,^33^. Our data also indicate that the combination treatment dramatically increases the intracellular ROS levels in THP-1 and U937 cells, whereas the highly significant increase (*p* < 0.001) in ROS following the combination treatment was blocked in the presence of N-acetyl-L-cysteine (4 mM NAC, an Intracellular ROS inhibitor) (Figure 4a) compared to without NAC-treated groups in both type of cells. To further investigate this effect, we next evaluated the THP-1 and U937 cells viability in the presence of NAC (Sigma-Aldrich) and observed that cell death was also dramatically increased in THP-1 and U937 cells after combination treatment (supporting information, Figure S10). It is well known that programmed cell death is induced mainly by the activation of a caspase-dependent pathway. Based on this knowledge, we intended to verify the activation of this pathway in response to combination treatment. We measured the caspase-3/7, and caspase-9 activities in THP-1 and U-937 cells, and our results show that caspases are activated after combination treatment (Figure 4b and 4c). To verify the caspase activity requirement, we incubated THP-1 and U937 cells with the pan-caspase inhibitor Z-VAD FMK (R & D systems, Minneapolis, MN, USA) and quantified cell death using the MTS assay. Treatment with Z-VAD FMK significantly (*p* < 0.05) attenuated the cell death induced by the combination treatment in both cancer cell lines (Figure 4d) compared to only combination-treated group. Hence, the Z-VAD FMK protected the cells from apoptosis induced by the combination treatment. To further investigate this mechanism, we determined whether DNA damage was induced by the combination treatment in the blood cancer cell lines, contributing to apoptosis induction. 8-Hydroxydeoxyguanosine (8-OHdG) is a ubiquitous marker of oxidative stress that is physiologically formed and enhanced by anticancer agents^34^,^35^. We found that the amount of DNA damage increased by approximately 3-fold following the combination treatment in THP-1 and U937 cells (Figure 4e). An early transient rupture in nuclear protein was recently shown to be required for apoptosis to proceed in cancer cells^36^, and this event results from the cleavage of poly (ADP-ribose) polymerase (PARP), which is catalyzed by caspase-3. Our results indicate that PARP cleavage increased by 47% and 60% after combination treatment in THP-1 and U937 cells, respectively, compared with the untreated control, whereas in the presence of the caspase inhibitor Z-VAD FMK, there was a decrease in PARP-1 cleavage by 20%–30% in both cell lines (Figure 4f and 4g).

### 2-DG and plasma combination treatment efficiently and selectively induce apoptosis in blood cancer cells through glucose deprivation

**Figure 5:**
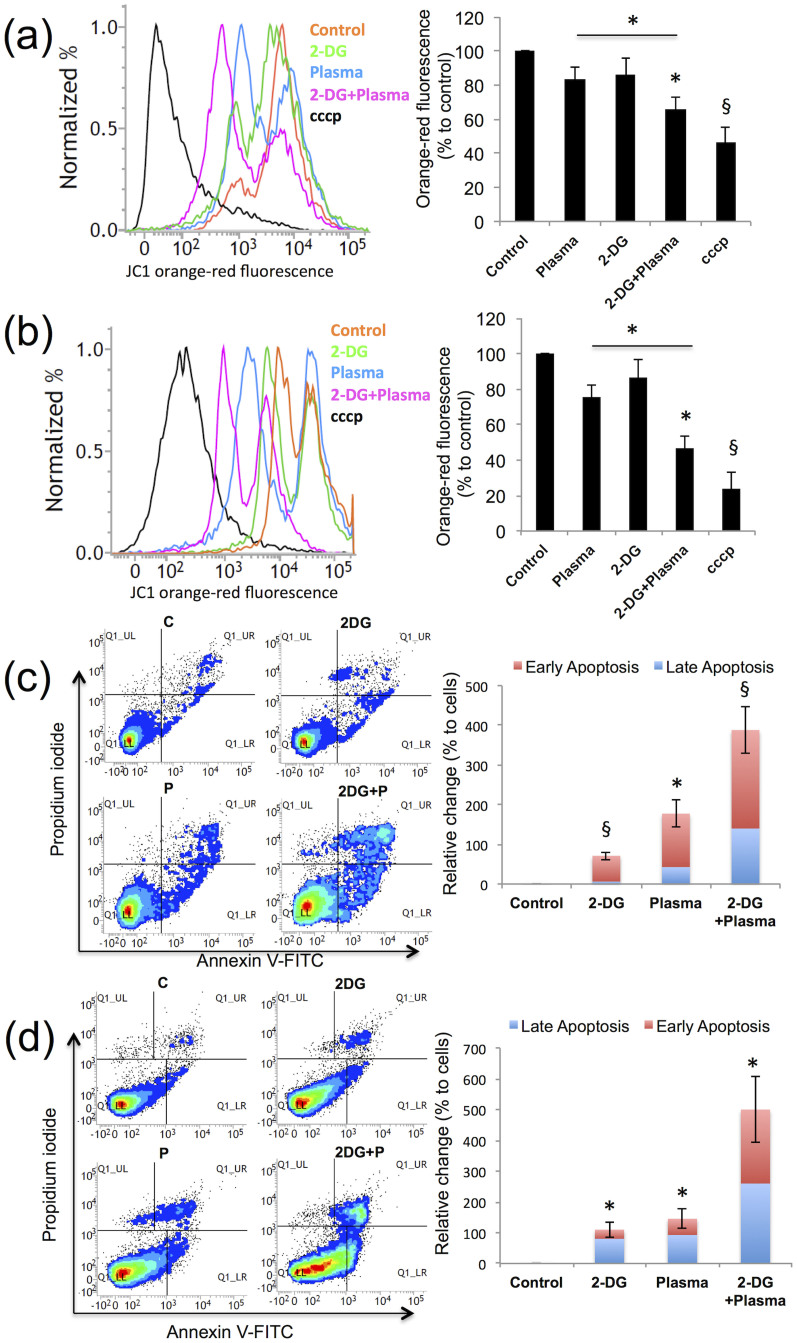
2-deoxy-D-glucose (2-DG) and plasma combination treatment efficiently and selectively induces apoptosis in blood cancer cells through glucose deprivation. (a-d) Apoptosis was analyzed in THP-1 and U937 cells following the combination treatment (1 mM 2-DG and 3 min plasma) using flow cytometry. (a, b) The mitochondrial membrane potential was measured using JC-1 cationic dye in THP-1 and U937 cells, respectively. (c, d) The apoptosis analysis of THP-1 and U937 cancer cells exposed to similar combination treatment (control denotes as C and plasma denotes as P), was conducted as a cytofluorimetric analysis of annexin V-FITC versus PI staining, respectively. Error bars designate the ± SD (n = 3). Student's *t*-test was performed, and the significance is indicated as * *p* < 0.05, § *p* < 0.01, and # *p* < 0.001.

The mitochondrial membrane potential (Δψm) is known to be a hallmark of apoptosis^37^. To verify the MMP change, THP-1 and U937 blood cancer cells were stained with the cationic dye JC-1, which accumulates in the inner mitochondrial membrane and will fluorescence red and green^38^. The proton ionophore Carbonyl cyanide 3-chlorophenylhydrazone (cccp) rapidly changes the MMP. Figure 5a and 5b shows the band shift phenomenon for the red fluorescence and the calculated percent intensity change in THP-1 and U937 cells, respectively, after the 2-DG and plasma treatment. The red fluorescence intensity was decreased by 30% to 40% (decrease in MMP) in both cell lines after the combination treatment. The graph shows >50% reduction in the MMP (50% increase in red fluorescence) in both cell lines when they were treated with a 2-DG and plasma combination treatment. To further characterize apoptosis, we stained THP-1 and U937 cells with Annexin V-FITC and PI. The combination treatment significantly (*p* < 0.05) increased the apoptotic population in THP-1 and U937, respectively, compared with the plasma alone-treated group (Figure 5c and 5d). Additionally, TUNEL staining was performed to visualize apoptotic cells^39^. Our TUNEL staining results show that both cell lines undergo apoptosis by nuclear damage (supporting information, Figure S11).

### 2-DG and plasma combination treatment induces apoptosis via the intrinsic pathway

**Figure 6:**
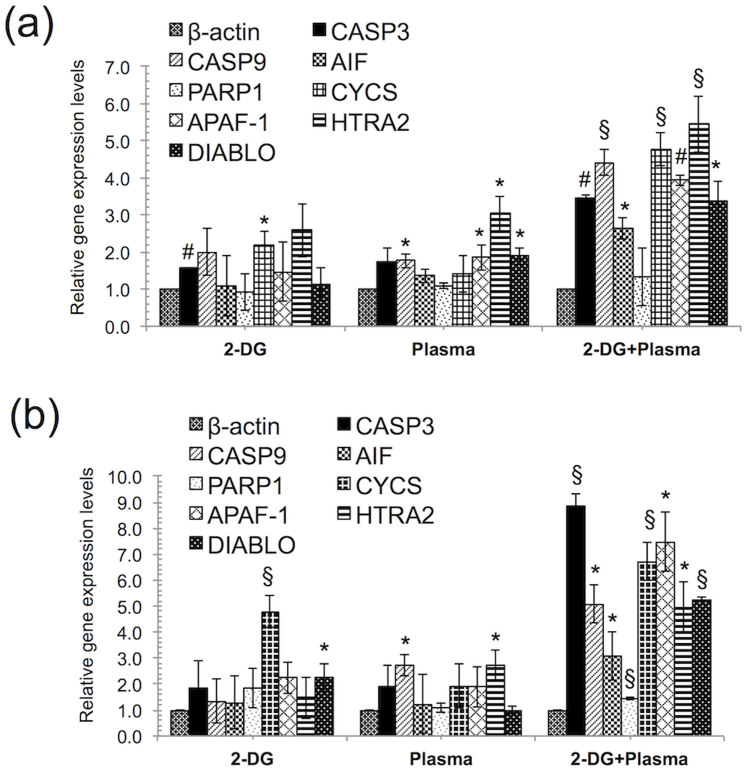
2-deoxy-D-glucose (2-DG) and plasma combination treatment induces apoptosis via the intrinsic apoptosis pathway. (a, b) The mRNA expression levels of related to intrinsic apoptosis pathway were quantified using real time RT–PCR in THP-1 and U937 cells, respectively after 24 h following the combination treatment (1 mM 2-DG and 3 min plasma). Error bars designate the ± SD (n = 3). Student's *t*-test was performed, and the significance is indicated as * *p* < 0.05, § *p* < 0.01, and # *p* < 0.001.

Next, to evaluate the apoptosis pathway induced by the plasma and 2-DG combination treatment, we studied the components that were mainly involved in mitochondrial-mediated apoptosis in blood cancer cells treated with the combination treatment. Generally, the mitochondrial-mediated apoptosis pathway is initiated by the release of apoptotic factors such as cytochrome c (cyt c), apoptosis-inducing factor (AIF), DIABLO (direct inhibitor of apoptosis protein (IAP)-binding protein), HTRA2 from the mitochondrial intermembrane space. The release of cyt c into the cytosol triggers caspase-3 activation through formation of the apoptosome complex, whereas DIABLO and HTRA2 promote caspase activation through neutralizing the inhibitory effects to the IAPs. We investigated the mRNA expression levels of mitochondrial apoptosis-related genes in THP-1 and U937 cells. We found that THP-1 and U937 cells showed higher expression of CASP3, CASP9, AIF, CYCS, APAF-1, HTRA2, and DIABLO gene expression levels (Figure 6a and 6b) with the combination treatment compared with the control. Interestingly, HTRA2 showed higher expression in THP-1 cells. HTRA2 belongs to the family of high temperature requirement protein A (HtrA) serine proteases that acts as a pro-apoptotic factor following release from the mitochondrial matrix through large nonselective pores (permeability transition pores, mPTPs) that can be opened by overproduction of ROS^40^. Once in the cytosol, HTRA2 promotes cell death by two different mechanisms. It can either bind to an inhibitor of apoptosis proteins (IAPs) via its amino terminal, reaper-related motif, or thus, induce caspase activity, or it can mediate caspase-independent death through its own protease activity. These data suggest that the combination treatment may induce apoptosis through both the caspase-dependent and independent pathway.

### 2-DG and plasma combination treatment is more effective than other drugs and conventional gamma radiation treatments

**Figure 7:**
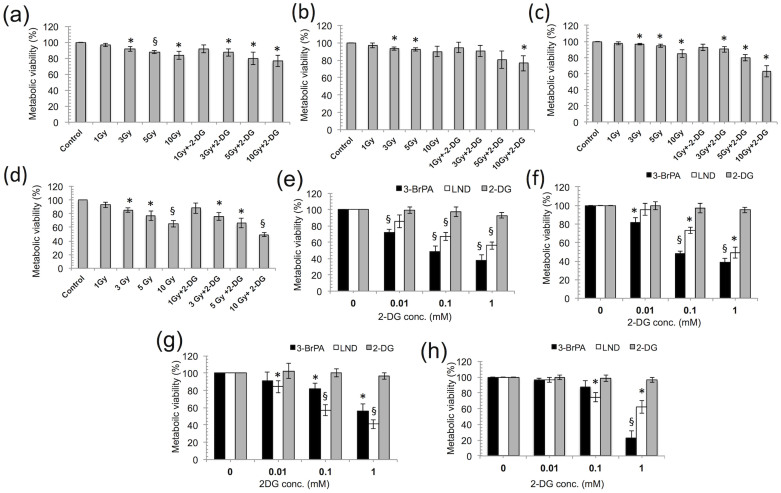
2-deoxy-D-glucose (2-DG) and plasma combination treatment is more effective than other drugs and conventional gamma radiation treatments. (a–d) Comparison of our combination treatment approach (2-DG and plasma) with conventional gamma radiation treatment at a dose of 1, 3, 5, and 10 Gy was conducted using the MTS assay on THP-1, U937, RAW264.7 and PBMCs, respectively at 24 h. (e–h) A comparison of 2-DG with 3-BrPA and LND (0.01, 0.1, and 1 mM) was conducted at 24 h, using the MTS assay on THP-1, U937, RAW264.7 and PBMCs, respectively. Error bars designate the ± SD (n = 3). Student's *t*-test was performed, and the significance was indicated by * *p* < 0.05, § *p* < 0.01, and # *p* < 0.001.

To determine the safety and efficacy of our combination approach (2-DG and plasma), we compared the 2-DG treatment with gamma radiation (GR). Note that, a low dose of GR is not effective, even when used in combination with 2-DG, in all of the blood cancer cells tested. We also observed an inhibitory effect of GR, alone or in combination with 2-DG (1 mM), on the normal counterparts in a dose-dependent fashion (Figure 7a, supporting information, Figure S12). This result indicates that short time incubation of 2-DG treatment with plasma is more effective than combination with gamma radiation. Conversely, when we compared the 2-DG toxicity with the toxicity of other available market drugs, such as 3-bromopyruvic acid (3-BrPA) and lonidamine (LND), we found that the toxicities of 3-BrPA and LND were greater than toxicity of 2-DG in the cancer cells and normal cells (Figure 7b). To summarize, plasma with 2-DG could be the safest approach for cancer and normal cells compared with the other related drugs or gamma radiation treatments.

## Discussion

In the present study, we provide evidence that a combination treatment of 2-DG and plasma synergistically induce the death of human blood cancer cells. Our previous study showed that jet plasma could induce ROS-dependent cell death in monocytic lymphoma^26^. The jet plasma significantly enhanced the inhibition of cell growth at only high doses (≥4 min), but the normal cells were also severely affected at these effective doses. Interestingly, targeting cellular metabolism is currently a novel approach for selectively killing cancer cells^41^,^42^,^43^. Recently, it was shown that 2-DG alone does not have a significant effect on tumor growth inhibition *in vivo*. However, the combined treatment of 2-DG with radiation treatments or anticancer chemical agents enhanced the cancer killing effects^44^,^45^. Based on these findings, we conducted the present study to find a mediator that could increase the efficiency and selectivity of plasma at low doses without affecting the normal counterparts. When blood cancer cells were treated with plasma and 2-DG, a glycolysis inhibitor^46^,^47^,^48^,^49^, we found that this combination treatment greatly improved the efficacy of plasma at low doses (≤3 min) (Figure 2). Combination treatment has an effect on both cancerous and non-cancerous PBMCs. Combination treatment significantly (*p* < 0.05) affect THP-1 and U937 cancer cells at all doses (1 mM 2-DG + 3 min plasma, 5 mM 2-DG + 1 min plasma, 5 mM 2-DG + 3 min plasma, 10 mM 2-DG + 1 min plasma and 10 mM 2-DG + 3 min plasma) except combination dose of 1 mM 2-DG and 1 min plasma. However, there was no significant effect (*p* > 0.05) on PBMCs with combination or 2-DG alone at lower doses such as <10 mM of 2-DG. Combination treatment showed significant effect (*p* < 0.05) only at high dose of 2-DG (10 mM) with plasma on PBMCs. We checked synergism of combination treatment by Chou and Talalay method. Chou introduced the concept of combination index (CI) for quantitative definition of synergism, additive effect, and antagonism. Synergism is basically a physicochemical action issue, not a statistical issue^31^. We determined synergism with CI values given by Chou and Talalay, not with p values. 2-DG and Plasma combined effect greater than each treatment alone does not necessarily indicate synergism. Sometimes this can be a result of additive effect or even a slight antagonism. Thus, if the combined effect is greater than each drug alone, it does not necessarily indicate synergism^30^. According to Chou and Talalay explanation for synergism determination, lower doses (1 mM 2-DG and 1 min plasma) show no or moderate synergism, however higher doses (10 mM 2-DG with 1 and 3 min plasma) have synergism against PBMCs. 2DG and plasma combination treatment shows synergism (CI < 0.7) against THP1 and U937 cells at all doses. In case of PBMCs combination treatments shows slight/moderate (CI > 0.7) or no synergism (CI > 1) at low doses of 2DG (<10 mM). However synergism (CI < 0.7) is showed by combination treatment only at high dose of 2DG (10 mM) against PBMCs.

It is worth mentioning that 2-DG weakens cancer cells by depleting the cell's energy (ATP) through the inhibition of the glycolysis pathway, affecting DNA repair activity and the production of ROS scavengers or antioxidants. Many reports showed that ROS play a main role in plasma-induced cancer cell apoptosis^50^,^33^. During treatment, the plasma induces the generation of a high level of ROS in cancer cells. It may be the reason that 2-DG pre-treated cells cannot handle this high level of ROS, and eventually the combination treatment can more preferentially and selectively kill cancer cells, as shown by the viability determination, flow cytometry and TUNEL labeling results (Figure 2 and 5, Figure S11). Earlier reports suggest that apoptosis can be induced by the intrinsic pathway (mitochondria-mediated) or by signaling from death receptors present on cell surface (extrinsic pathway)^51^,^52^,^53^. It has already been well established that ROS mainly mediate the mitochondrial apoptosis pathway^54^,^55^,^56^. The combination treatment of 2-DG and plasma also reduces the mitochondrial membrane potential, the release of cytochrome c into the cytosol and activates the caspases (Figure 4 and 5) that catalyze PARP activity and eventually the DNA fragmentation contributing to apoptosis. Furthermore, the DIABLO, AIF gene expression level was also shown to increase during the apoptosis following the combination treatment. AIF can be released from the mitochondria on outer mitochondrial membrane permeabilization, and shift to the nucleus to promote nuclear condensation and ultimately DNA damage. Also, DIABLO and HTRA2 can trigger apoptosis by counteract the activity of IAPs by a caspase-independent path. These data support our proposal that the combination treatment of 2-DG with plasma induces apoptosis via the caspase-dependent and independent pathways (supporting information, Figure S13).

In conclusion, our results may help to improve the efficacy of plasma treatment using 2-DG and may contribute to future plasma therapy strategies. Additionally, we also propose that a decrease in the intracellular ATP level is a requisite for the apoptotic cell death process in cancer cells. Normal cells are less affected by the combination treatment because they are less dependent on glucose consumption and have robust antioxidant machinery. Moreover, when comparing 2-DG with other commercial drugs (3-bromopyruvic acid and lonidamine) and conventional gamma radiation treatment; these treatments appear to be severely toxic in the normal counterparts in a dose-dependent manner. Finally, we conclude that the combination of 2-DG and plasma may represent a novel strategy for cancer therapeutic treatment and may overcome the cytotoxic effect of conventional chemotherapeutic agents and radiation treatment.

## Methods

### Cell culture

Three blood cancer cell lines were used in this study. The U937 cell line (human monocyte lymphoma) was purchased from the Korean cell line bank (SNU, Seoul). The THP-1 (human leukemic monocytic cell) and RAW264.7 (mouse leukemic monocyte macrophage) cell lines were kind gifts given by Prof. Sang Soo Lee, Hallym University, Chuncheon, Korea. Peripheral blood mononuclear cells (PBMCs, normal cells) were purchased from Lonza, USA. U937 and THP-1 cells were maintained in RPMI-1640 medium (Hyclone, USA) supplemented with 10% fetal bovine serum. In case of RAW264.7 cells, we used high glucose DMEM (Hyclone, USA) medium supplemented with 10% fetal bovine serum including antibiotics. PBMCs were cultured in mononuclear medium (Lonza, USA). All stock cultures were maintained in 5% CO_2_ and humidified air at 37°C.

### 2-deoxy-D-glucose and non-thermal jet plasma treatment

The glycolysis inhibitor 2-DG was obtained from Sigma-Aldrich, Korea and used without further purification. Stock solutions of 2-DG were dissolved in PBS and the required volume was added directly to achieve the desired final concentration. 2-DG was added for 4 h prior to each experiment. Cells in culture medium were plated onto a 24-well tissue culture plate and incubated at 37°C in a humidified 5% CO_2_ atmosphere. Cells were exposed to plasma for 1 and 3 min at 6 mm distance.

### Cell survival assays

Blood cancer and normal cells (2 × 10^5^) were treated with different concentrations of 2-DG in medium and then treated with plasma (1 and 3 min). After the indicated times (24, 48 and 72 h), cell viability was measured using the CellTiter 96® Aqueous One Solution Cell Proliferation Assay Kit (Promega, Korea) as previously described^26^. The results are expressed as percentage (%) viability, which is directly proportional to the number of metabolically active cells and was calculated as previously described^16^,^57^. We also counted number of cells using trypan blue exclusion dye (0.1%, Sigma Aldrich, Korea) and a hemocytometer (Marienfeld, Germany) for quantitative comparison.

### Intracellular ROS detection

To determinethe ROS level inside the cells, cells were harvested after the combination treatment (2-DG and plasma) and incubated with 10 μM 2′,7′-dichlorodihydrofluorescein diacetate (H_2_DCFDA; Molecular Probes) for 30 min. The DCF fluorescence was quantified at 485/540 nm using a plate reader (Synergy HT, Biotek). To investigate the role of ROS in cell death, the cells were pre-incubated with 4 mM NAC, a ROS inhibitor.

### Metabolic energy marker measurements

Glucose consumption and the lactate level were calculated using the Glucose (HK) Assay Kit (Sigma-Aldrich) and the EnzyChrom™ Lactate Assay Kit (Bioassay systems). Intracellular ATP levels were measured using a commercially available EnzyLight^TM^ ATP assay kit (Bioassays Systems, CA). The ATP levels of the treated cells were normalized to the ATP levels of the untreated cells. The rate of oxygen consumption and the extracellular acidification rate were evaluated using a real time Seahorse XF24 Analyzer (Seahorse Bioscience, North Billerica, MA, USA) as previously described^58^. Briefly, 2 × 10^4^ cancer cells were seeded at 24 h before the 2-DG and plasma treatment. Before beginning the measurements, the cells were placed in a phenol red free DMEM medium (supplemented with 25 mM glucose, 2 mM glutamine, and 1 mM sodium pyruvate, and without serum) and pre-incubated for 1 h at 37°C in atmospheric CO_2_. To determine the OCR values, these values were normalized for the protein content of each sample.

### DNA damage assay

After the combination treatment, the amount of 8-hydroxy-2′-deoxyguanosine (8-OHdG), a by-product of DNA damage, was determined in THP-1 and U937 cells using an ELISA based method according to the OxiSelect™ Oxidative DNA Damage ELISA Kit (Cell Biolabs, Inc.) protocol.

### Terminal deoxynucleotidyltransferase dUTP nick end labeling (TUNEL) assay

TUNEL staining was performed to label DNA strand breaks for the detection of apoptotic cells. All of the detection steps were performed according to the manufacturer's, protocol (APO-BrdU™ TUNEL Assay Kit - with Alexa Fluor® 488 Anti-BrdU Kit, Molecular probes, Invitrogen).

### PARP (Poly (ADP-ribose) polymerase) cleavage assay

Quantitative measurement of the 89 kDa fragment (cleaved fragment) of human PARP-1 was conducted in THP-1 and U937 cells using an ELISA method provided in the Cleaved PARP-1 In-Cell ELISA Kit (Abcam, Korea).

### Analysis of cell apoptosis markers

To observe the changes in the mitochondrial membrane potential resulting from the combination treatment of 2-DG and plasma, JC-1 staining was performed on treated blood cancer cells using the MitoProbe JC-1 assay kit (Invitrogen, USA) as previously described^59^,^60^. Caspase activities were measured using the Caspase-Glo® 3/7 assay kit, and Caspase-Glo® 9 assay kit (Promega) on a white 96-well flat-bottom microplate according to the manufacturer's instructions. To verify caspase-dependent cell death, we pre-incubated the cells with Z–VAD FMK, a pan-caspase inhibitor, for a viability analysis before 2 h of combination treatment. Next, to further verify apoptosis, the treated cells were subjected to a FACS analysis. Briefly, at 24 h, cells were harvested and stained with annexin V-FITC/PI using the Annexin V: FITC Apoptosis Detection Kit I (BD Biosciences, Seoul, Korea) and directly analyzed using a flow cytometer (BD FACSVerse, NJ, USA) and the FACS suite software.

### Real-time reverse transcriptase-polymerase chain reaction (real-time RT-PCR)

Cells total RNA was isolated using the Trizol reagent (Invitrogen) and cDNA was synthesized using Superscript II reverse transcriptase kit (Invitrogen). Significant changes in the mRNA expression of the genes of interest were calculated by means of real-time RT-PCR. Real-time PCR was performed on a CFX96™ Real-Time System with a, BioRad machine with the IQ SYBR Green Supermix (BD Biosciences). The primer assays used in this study are listed in supporting information, Table S2. The PCR conditions consisted of a 10 min hot start at 95°C, followed by 40 cycles of 10–15 s at 95°C and 30–60 s for 1 min optimized temperature. The levels of gene expression relative to β-actin were determined as described by RT^2^ qPCR Primer Assay Handbook.

### Comparison of 2-DG with other available market drugs and gamma radiation

To evaluate the toxicity level of toxicity of 2-DG, we compared our samples with other available market drugs (3-BrPA and LND), which are also known as glycolytic inhibitors. Briefly, prior to the experiment, cells (2 × 10^5^) were seeded onto 24 well culture plate for 24 h and treated with different concentrations of 2-DG, 3-BrPA and LND (0.01, 0.1, and 1 mM) and further incubated for 24 h. Approximately 3 h before the desired time, MTS solution was added to each well, the absorbance readings were measured at 490 nm and the viability results were calculated as previously described. To compare our treatment approach with gamma radiation treatment, cells were exposed to radiation alone or in combination with 2-DG (1 mM), using a 137Cs c-ray source (Atomic Energy of Canada, Ltd, Mississauga, ON, Canada) at a dose rate of 1,3,5, and 10 Gy/min. The viability of the treated cells was assessed 24 h post-treatment using the MTS assay as previously mentioned.

### Statistical analyses

All result values were expressed as the mean ± standard deviation (S.D.) of four independent tests. Statistical analysis was performed using Student's *t*-test. Statistical significance was recognized at * *p* < 0.05, § *p* < 0.01, and # *p* < 0.001.

## Supplementary Material

Supplementary Information Supporting information
