# Supplementary material for: Non-thermal plasma with 2-deoxy-D-glucose synergistically induces cell death by targeting glycolysis in blood cancer cells
Source: Sci Rep. 2015 Mar 4;5:8726. doi: 10.1038/srep08726 (PMC5390089; doi:10.1038/srep08726)
Supplement: Supplementary Information — Supporting information [file srep08726-s1.pdf]

Supporting Information

Non-thermal plasma with 2-deoxy-D-glucose synergistically induces cell death by targeting glycolysis in blood cancer cells

Neha Kaushik,[†1] Su Jae Lee,[2] Tae Gyu Choi[3], Ku Youn Baik,[1] Han Sup Uhm,[1] Chung Hyeok Kim,[4] Nagendra Kumar Kaushik,[1†]* Eun Ha Choi [1]*

[1]*Plasma Bioscience Research Center, Kwangwoon University, Seoul 139-701, Korea*
[2]*Laboratory of Molecular Biochemistry, Department of Life Science, Hanyang University, Seoul 133-791, Korea.*
[3]*School of medicine, Department of Biochemistry and Molecular Biology, Kyunghee University, Seoul 130-701, Korea*
[4]*Institute of Information Technology, Kwangwoon University, 447-1, Seoul 139-701, Korea*

email: kaushik.nagendra@kw.ac.kr and ehchoi@kw.ac.kr

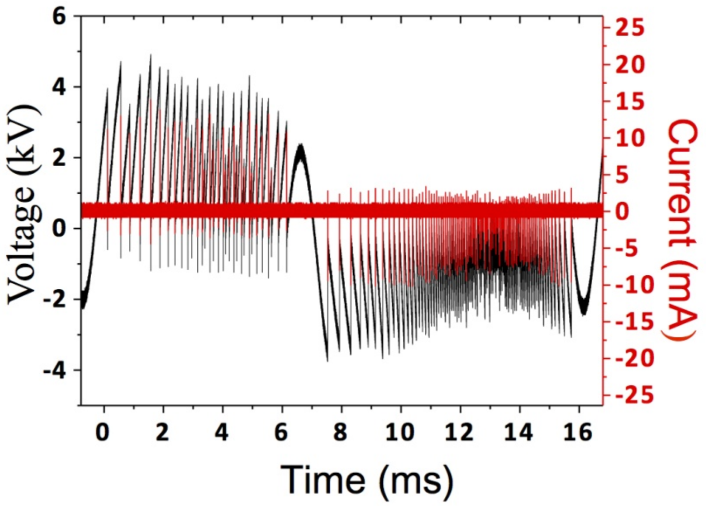

Figure S1. Voltage and current waveform of the plasma device (Sci Rep.,5,8587/Doi:10.1038/srep08587).

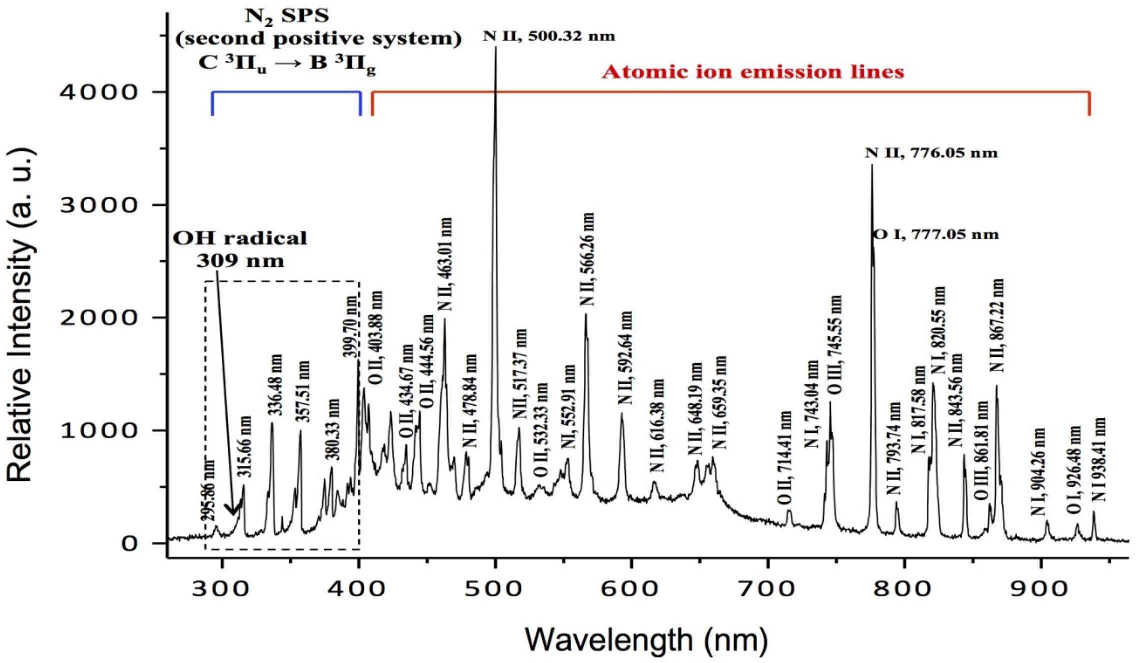

Figure S2. Optical emission spectra (OES) of the plasma device (Sci Rep.,5,8587/Doi:10.1038/srep08587).

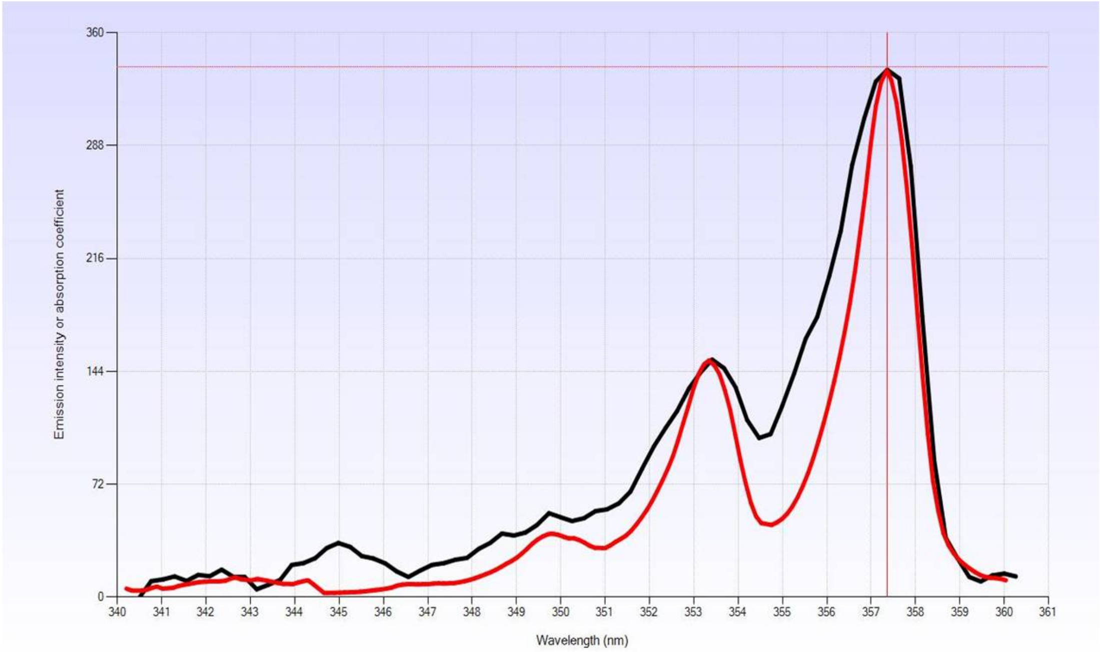

Figure S3. Measurement of gas temperature of the plasma device.

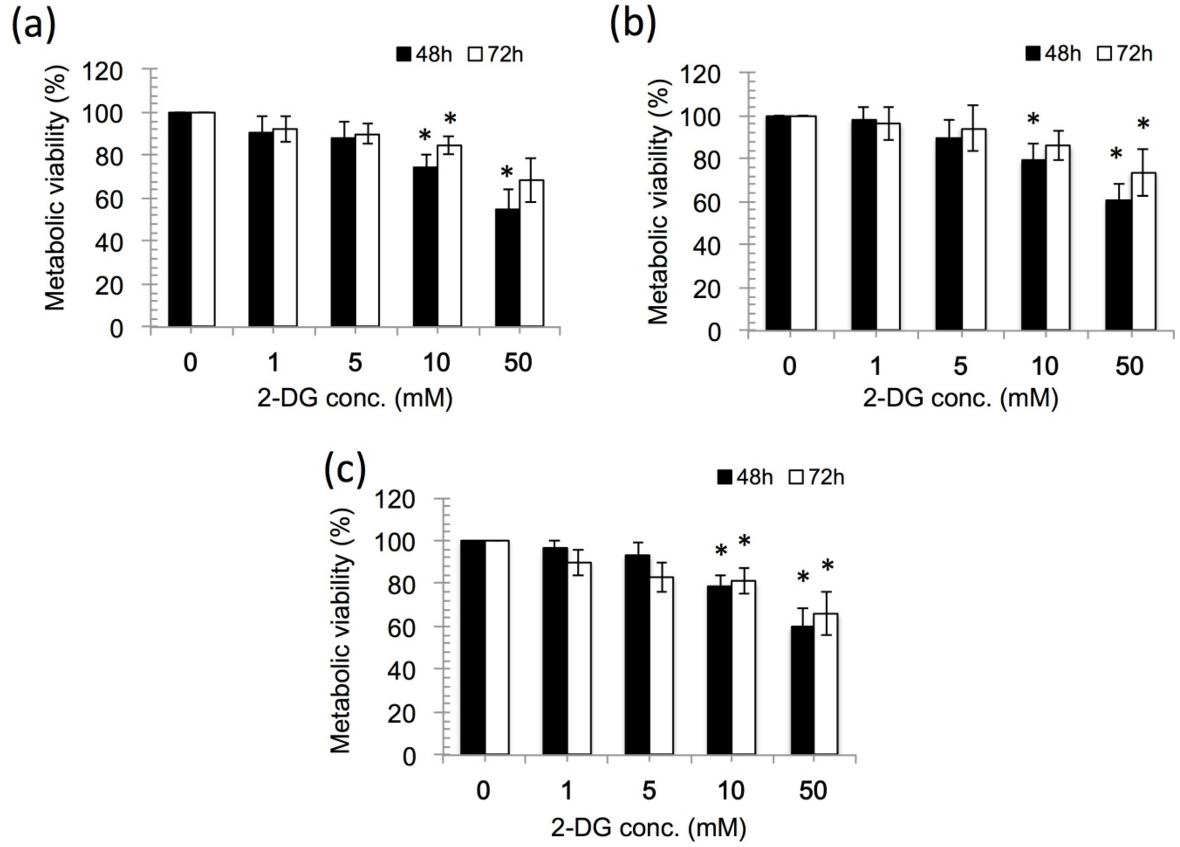

Figure S4. Effect of various concentrations of 2-deoxy-D-glucose (2-DG) alone, on blood cancer cells. Metabolic viability of (**a**) THP-1, (**b**) U937, and (**c**) RAW264.7 at 48 and 72 hours. The results were calculated as the percentage of viable cells and presented as the mean ± SD (n=3). Student's *t*-test was performed, and the significance is indicated as * $p<0.05$, § $p<0.01$, and # $p<0.001$.

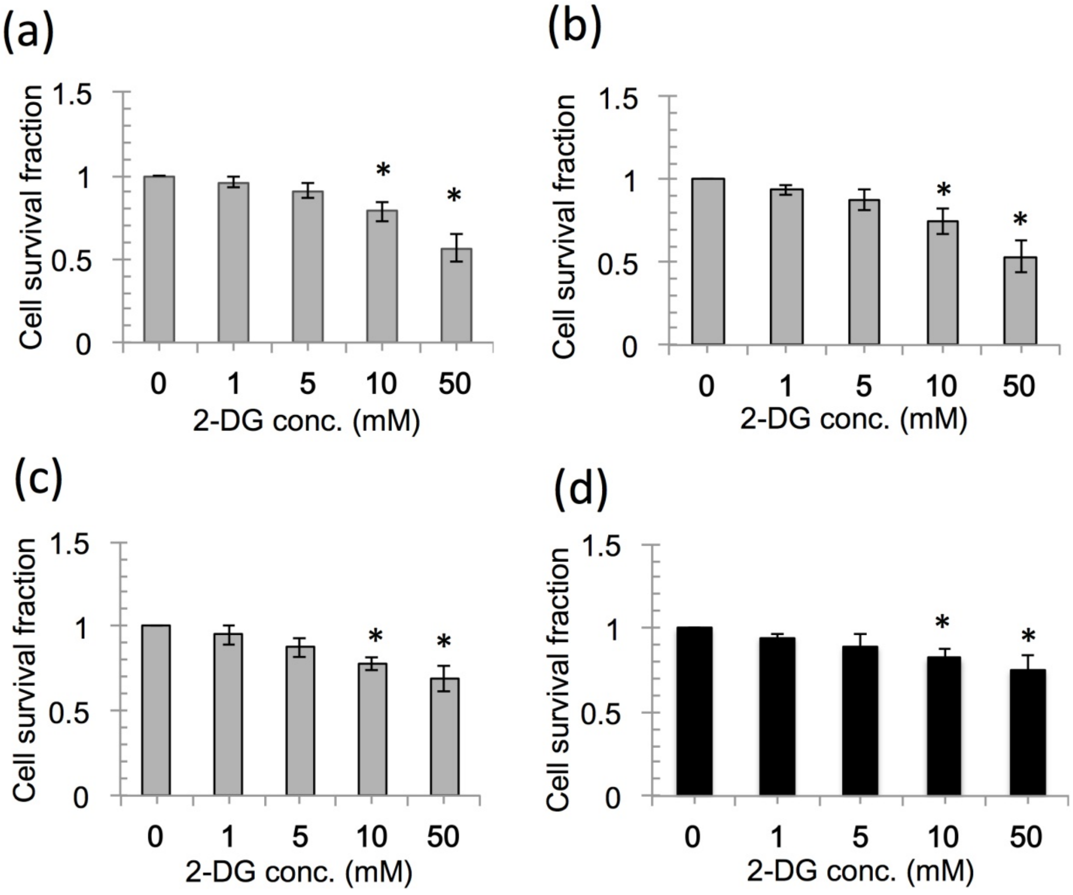

Figure S5. Cell count of (**a**) THP-1, (**b**) U937, (**c**) RAW264.7 and (**d**) PBMCs treated with different concentrations of 2-deoxy-D-glucose following 24 hour. The results were calculated as the percentage of viable cells and presented as the mean ± SD (n=3). Student's *t*-test was performed and the significance is indicated as * p<0.05, § p<0.01, and # p<0.001.

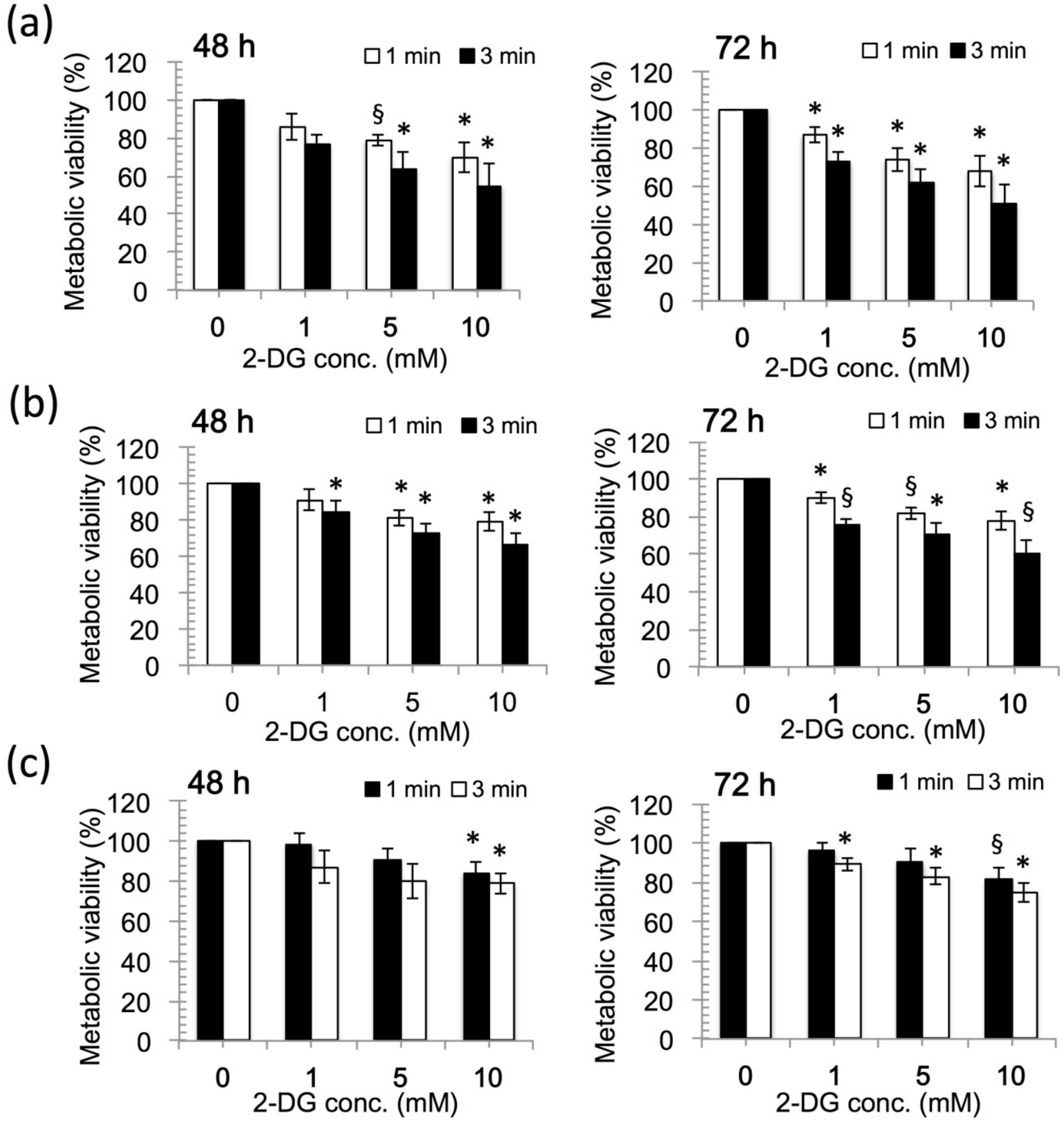

Figure S6. Response of 2-DG and plasma combination treatment on blood cancer cells. Metabolic viability of (**a**) THP-1, (**b**) U937 and (**c**) RAW264.7 following 48 and 72 hours using MTS assay. The results were calculated as the percentage of viable cells and presented as the mean ± SD (n=3). Student's *t*-test was performed, and the significance is indicated as * p<0.05, § p<0.01, and # p<0.001.

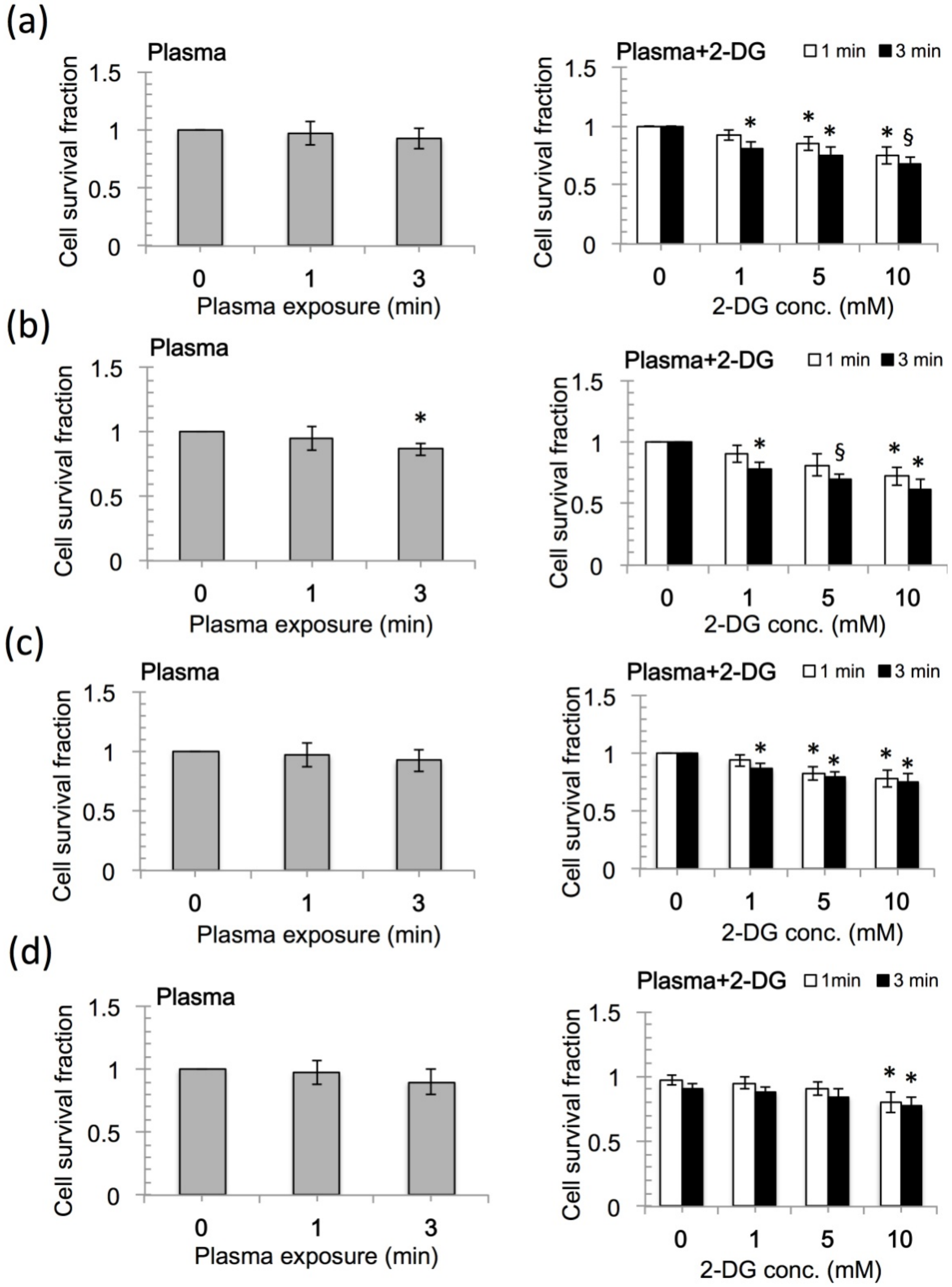

Figure S7. Cell count of (**a**) THP-1, (**b**) U937, (**c**) RAW264.7 and (**d**) PBMCs treated with 2-DG and plasma combination treatments following 24 hour. The results were calculated as the percentage of viable cells and presented as the mean ± SD (n=3). Student's *t*-test was performed, and the significance is indicated as * p<0.05, § p<0.01, and # p<0.001.

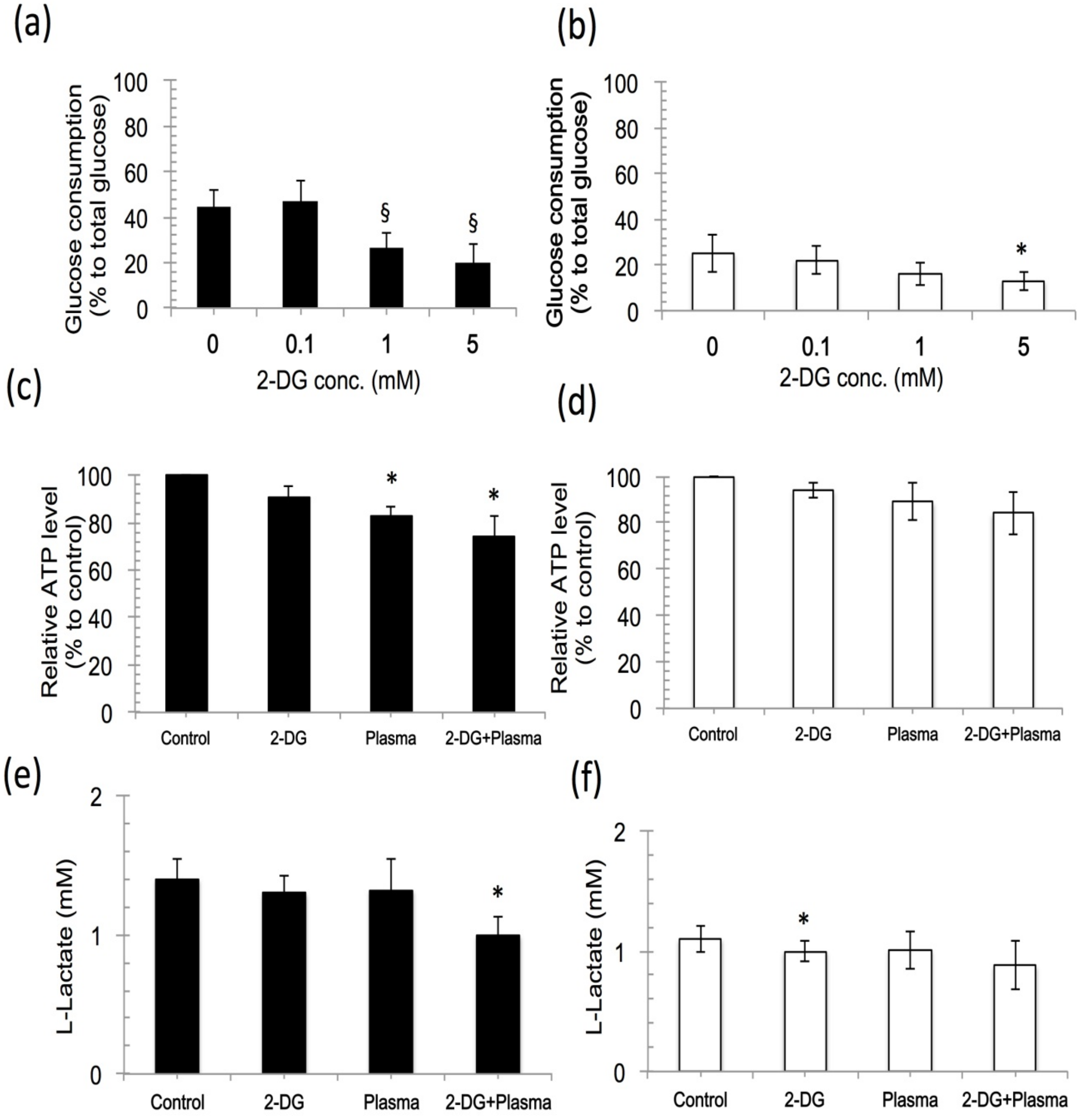

Figure S8. Metabolic alterations induced by 2-DG (1 mM) and plasma (3 min) combination treatment in blood cancer and normal cells. (**a, b**) Glucose consumption was examined at various concentrations of 2-DG (0.1, 1, and 5 mM) in RAW264.7 and PBMCs cells cultured in RPMI-1640 medium, respectively. (**c, d**) Cellular ATP levels were measured in RAW264.7 and PBMCs cells, respectively. (**e, f**) Lactate production was monitored in RAW264.7 and PBMCs cells cultured in RPMI-1640 medium, respectively. Error bars designate the ± SD (n=3). Student's *t*-test was performed, and the significance is indicated as * $p<0.05$, § $p<0.01$, and # $p<0.001$.

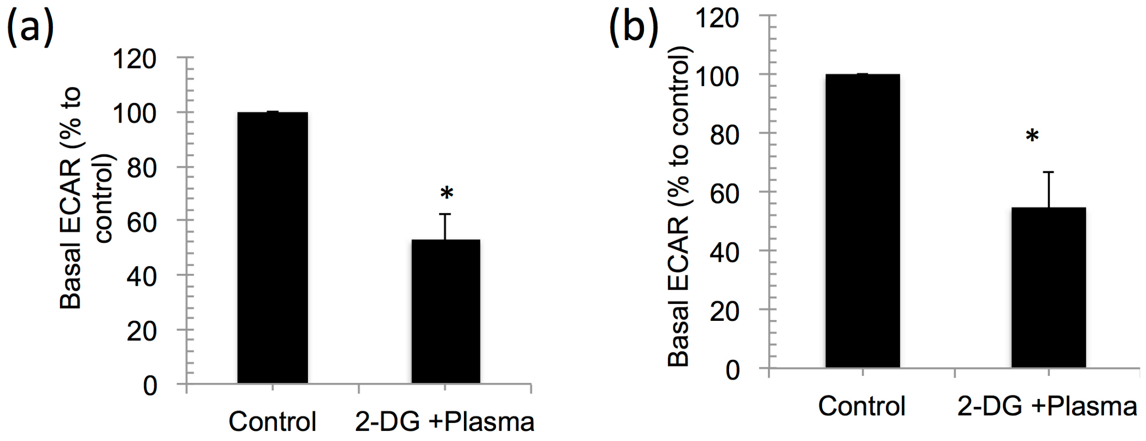

Figure S9. The ECAR in (a) THP-1 and (b) U937 cells following a 24 h combination treatment were determined using a seahorse XF24 analyzer. The ECAR, a measurement of lactate production/glycolysis, following treatment represents the acidification rate associated with glycolytic activity in THP-1 and U937 cancer cell lines, respectively. Error bars designate the ± SD (n=2). Student's t-test was performed, and the significance is indicated as * p<0.05, § p<0.01, and # p<0.001.

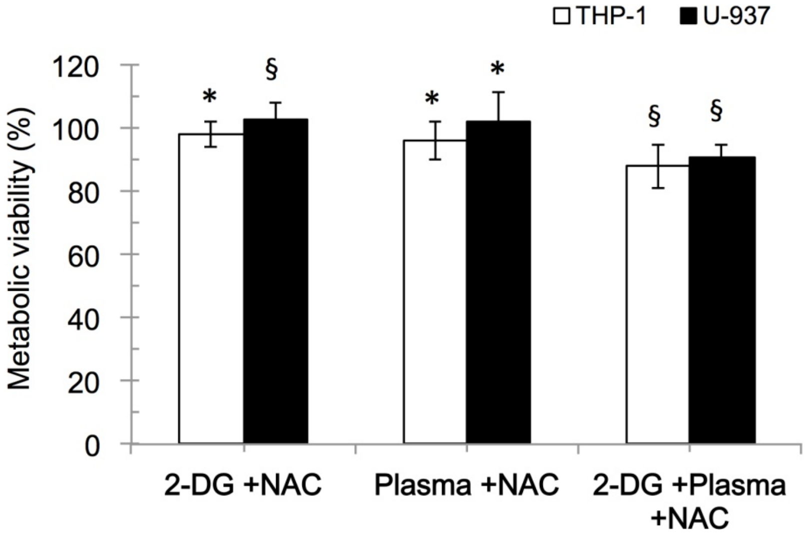

Figure S10. Metabolic viability of THP-1 and U937 cells in the presence of NAC (intracellular ROS inhibitor), following 24 h of combination treatment using MTS assay. Error bars designate the ± SD (n=3). Student's *t*-test was performed between NAC-treated and NAC-untreated groups and the significance is indicated as * p<0.05, § p<0.01, and # p<0.001.

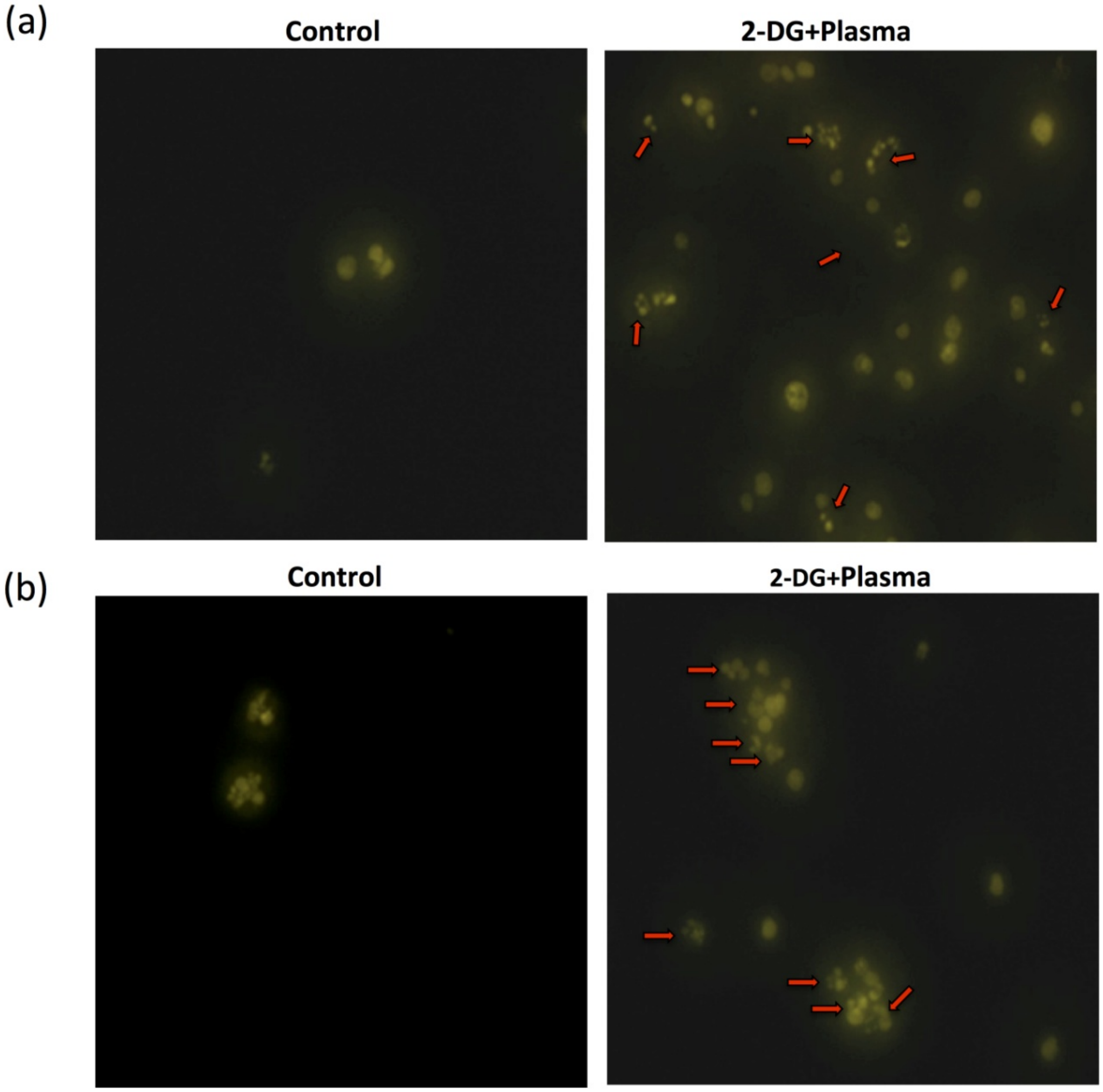

Figure S11. TUNEL staining was performed on (**a**) THP-1 and (**b**) U937 cells, followed by 2-DG (1 mM) and plasma (3 min) combination treatment. This staining discriminates the cells that have undergone apoptotic mechanisms. Cells were grown on the coverslips and treated with following combination treatment and staining was performed according to manufacturer's protocol (APO-BrdU™ TUNEL Assay Kit - with Alexa Fluor® 488 Anti-BrdU Kit, Molecular probes, Invitrogen).

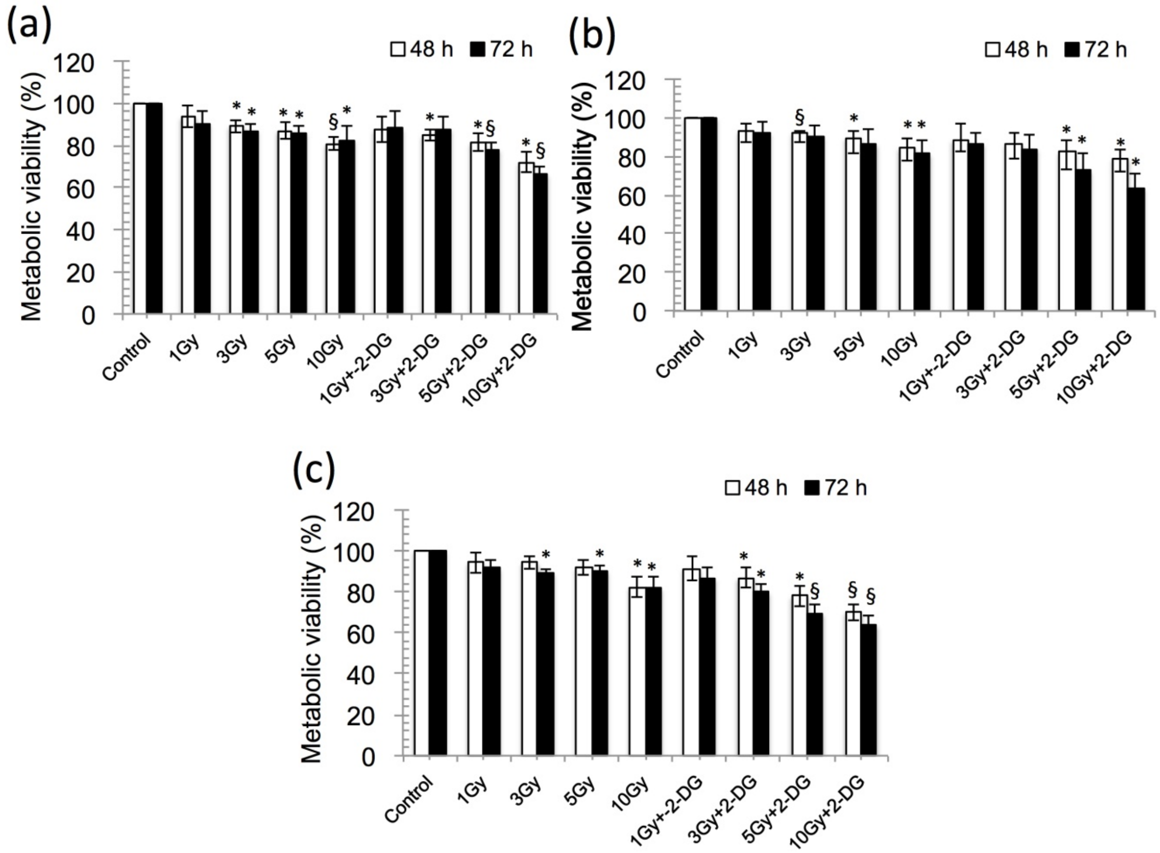

Figure S12. Comparison of our combination treatment approach (2-DG and plasma) with conventional gamma radiation treatment at a dose of 1, 3, 5, and 10 Gy was conducted using the MTS assay on (**a**) THP-1, (**b**) U937 and (**c**) RAW264.7 respectively at 48 and 72 h. Error bars designate the ± SD (n=3). Student's *t*-test was performed, and the significance is indicated as * p<0.05, § p<0.01, and # p<0.001.

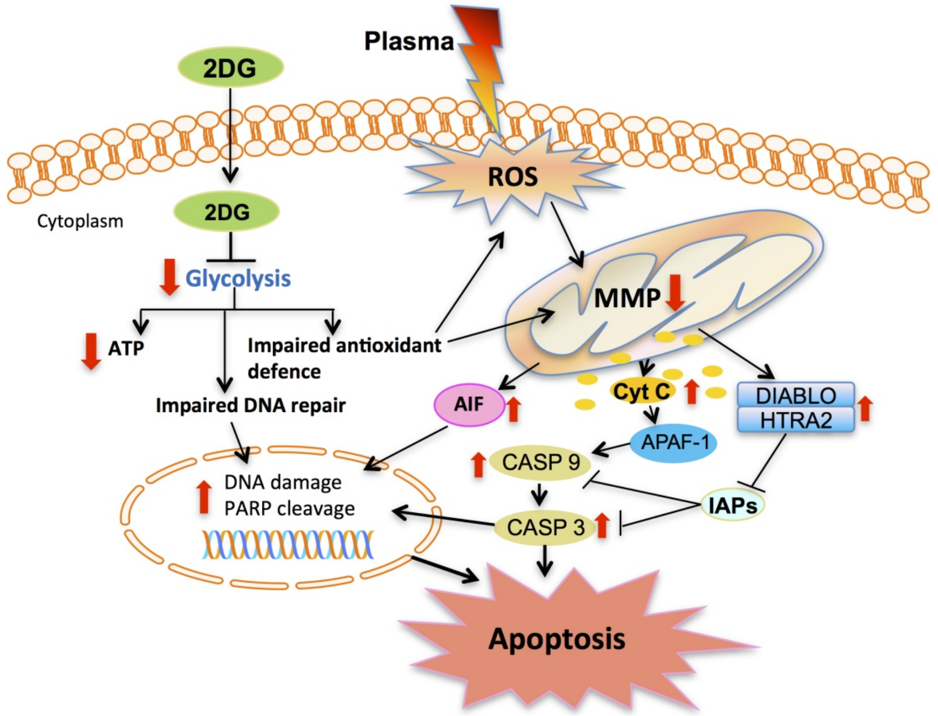

Figure S13. Proposed mechanism of 2-deoxy-D-glucose with non-thermal plasma that induce the synergistic inhibition effect in blood cancer cells. 2-deoxy-D-glucose (2-DG) enters inside the cell cytoplasm through cell membrane and forms 2-DG-6-phosphate by hexokinase phosphorylation, which is the first step of glycolysis pathway. This 2-DG-6-phosphate cannot further metabolize and trapped in the cytosol and eventually inhibits glycolysis. This result in decrease of cellular ATP, lactate production, impaired antioxidant machinery and DNA repair activity in cancer cells and consequently, cells become weak. However, plasma can generate oxidative stress through ROS (reactive oxygen species), which leads to the change in mitochondrial membrane potential (MMP) that triggers mitochondrial mediated intrinsic apoptosis pathway. This pathway subsequently leads to the release of cytochrome c (Cyt c), apoptosis-inducing factor (AIF), direct inhibitor of apoptosis protein (IAP)-binding protein with low PI (DIABLO), and HTRA serine peptidase 2 (HTRA2). On release to the cytosol, cytochrome c triggers caspase 3 activation through the formation of apoptosome complex and catalyse the PARP1 activity and leads to the DNA damage contributing to caspase-dependent apoptosis, whereas DIABLO and HTRA2 triggers caspase 3 activation by neutralizing the activity of IAPs (inhibitor of apoptosis protein). Similarly, AIF can be released from the mitochondria on outer mitochondrial membrane permeabilization, and move to the nucleus to contribute DNA damage or fragmentation. Taken together, 2-DG and plasma combination synergistically induced mitochondrial intrinsic apoptosis in cancer cells that can be either caspase-dependent or independent.

Table S1. This table provides the Combination Index (CI) value that was obtained when combining 2-DG with plasma at different doses on blood cancer and normal cells, using the Chou-Talalay method. CI < 1: Synergy; CI=1: Additive effect; CI > 1: Antagonism.

Treatments	THP-1	U937	RAW264.7	PBMCs
1 mM 2DG + 1 min plasma	0.50	0.59	3.99	2.8
1 mM 2DG + 3 Min Plasma	0.40	0.55	1.59	0.71
5 mM 2DG + 1 min Plasma	0.51	0.56	1.10	0.79
5 mM 2DG + 3 min plasma	0.50	0.39	1.47	1.01
10 mM 2DG + 1 min plasma	0.68	0.75	0.77	0.64
10 mM 2DG + 3 min plasma	0.51	0.34	0.96	0.58

Table S2. The list of RT^2 qPCR Primer Assays (Qiagen, USA) used for real-time PCR analysis.

S.N.	Catalog No.	Product Description	Gene code
1.	PPH00073G	RT^2 qPCR Primer Assay for ACTB	Beta Actin (Human)
2.	PPH00107C	RT^2 qPCR Primer Assay for CASP3	CASP3 (Human)
3.	PPH00353B	RT^2 qPCR Primer Assay for CASP9	CASP9 (Human)
4.	PPH00686B	RT^2 qPCR Primer Assay for PARP1	PARP1 (Human)
5.	PPH01037A	RT^2 qPCR Primer Assay for AIFM1	AIFM1 (Human)
6.	PPH00752A	RT^2 qPCR Primer Assay for APAF1	APAF-1 (Human)
7.	PPH20675F	RT^2 qPCR Primer Assay for CYCS	CYCS (Human)
8.	PPH11316A	RT^2 qPCR Primer Assay for HTRA2	HTRA2 (Human)
9.	PPH05797A	RT^2 qPCR Primer Assay for DIABLO	DIABLO (Human)
